# Gender and ethnic health disparities among the elderly in rural Guangxi, China: estimating quality-adjusted life expectancy

**DOI:** 10.3402/gha.v9.32261

**Published:** 2016-11-03

**Authors:** Tai Zhang, Wuxiang Shi, Zhaoquan Huang, Dong Gao, Zhenyou Guo, Virasakdi Chongsuvivatwong

**Affiliations:** 1 Epidemiology and Biostatistics Unit, Faculty of Public Health, Dali University, Dali, China; 2 Epidemiology Unit, Faculty of Medicine, Prince of Songkla University, Songkhla, Thailand; 3 Health Management Unit, Faculty of Humanities and Management, Guilin Medical University, Guilin, China

**Keywords:** gender, ethnicity, inequality in health, quality-adjusted life expectancy, rural elderly

## Abstract

**Background:**

Ethnic health inequalities for males and females among the elderly have not yet been verified in multicultural societies in developing countries. The aim of this study was to assess the extent of disparities in health expectancy among the elderly from different ethnic groups using quality-adjusted life expectancy.

**Design:**

A cross-sectional community-based survey was conducted. A total of 6,511 rural elderly individuals aged ≥60 years were selected from eight different ethnic groups in the Guangxi Zhuang Autonomous Region of China and assessed for health-related quality of life (HRQoL). The HRQoL utility value was combined with life expectancy at age 60 years (LE60) data by using Sullivan's method to estimate quality-adjusted life expectancy at age 60 years (QALE_60_) and loss in quality-adjusted life years (QALYs) for each group.

**Results:**

Overall, LE_60_ and QALE_60_ for all ethnic groups were 20.9 and 18.0 years in men, respectively, and 24.2 and 20.3 years in women. The maximum gap in QALE_60_ between ethnic groups was 3.3 years in males and 4.6 years in females. The average loss in QALY was 2.9 years for men and 3.8 years for women. The correlation coefficient between LE_60_ and QALY lost was −0.53 in males and 0.12 in females.

**Conclusion:**

Women live longer than men, but they suffer more; men have a shorter life expectancy, but those who live longer are healthier. Attempts should be made to reduce suffering in the female elderly and improve longevity for men. Certain ethnic groups had low levels of QALE, needing special attention to improve their lifestyle and access to health care.

## Introduction

Global trends in population aging are experiencing a rapid and unprecedented rise in the 21st century (1), and problems related to aging pose significant social challenges around the world. In China, there have been 200 million people aged 60 years and older since 2013, accounting for 14.9% of the total population (2). By 2053, it is predicted that this number will surpass 480 million and account for 34.9% of the total population (3). Currently, over 50% of the elderly are still living in rural China, which has become a top health issue and challenge for policy makers.

Health expectancy (HE) estimates the average expectation of life years lived in various health states into a single summary measure (4). Quality-adjusted life expectancy (QALE) is an HE measure that combines health-related quality of life (HRQoL) with life expectancy (LE) to yield a single summary score in expected years of life (5). Because HRQoL differs across different stages of life, calculating LE adjusted by HRQoL provides a more complete measure for assessing overall health (5). Consequently, QALE is a more sensitive and robust indicator for measuring population health status than LE or health-adjusted life expectancy (HALE) that is based on binary variables of health status (6). QALE is better for the elderly because it can capture both the quantity and quality of life dimensions of health by combining information on mortality with HRQoL. Consequently, this indicator is especially useful to reflect the overall health status of communities with rapidly increasing population aging and high disability rates.

To date, QALE has been widely used for monitoring the status of population health, predicting future health service needs, evaluating health programs, setting the age of retirement in a sensitive way, and planning for pension systems (7). Comparisons of QALE in different populations can be used to evaluate the performance of different health systems and to identify the determinants of health inequalities in subgroups (8). In China, although HRQoL data based on the European Quality of Life five-dimension three-level (EQ-5D-3L) questionnaire for the general population have been collected by the National Health Service Survey since 2008, QALE has not yet been used in routine health surveillance because the Chinese EQ-5D value sets only became available recently (9).

Ethnicity and gender are important determinants of the health status of a population. In gerontological research, however, the dominant approaches for assessing health status in the elderly have tended to treat them as a homogeneous group and have ignored differences based on gender and ethnicity (10). Previous studies have also revealed that HALE differs by gender and ethnicity. In general, women have a longer LE than that of men (11, 12) but are more likely to suffer problems such as depression (13), loneliness, and empty-nest syndrome in late life (14). Contrarily, men tend to report better quality of life (15). As a result, gender differences in HALE among older adults were inconsistent in previous studies. For example, there was no gender difference in HALE among older adults in Singapore (16). Little is known about the gender difference in HALE in China.

Recently, a few studies have observed race- or ethnicity-based inequalities in HE among populations from different cultural backgrounds (17). However, these studies of racial inequalities in HE focused on differentials between whites and other races in the United States (12, 18–20). The findings were that whites enjoyed more years in good health compared to any other ethnicity, but the gap decreased at older ages (12). Another study found that blacks had lower HALE than non-blacks. Race-based inequalities were larger among lower-income groups after adjustment for socioeconomic status (12). In Singapore, elderly Chinese had higher HALE compared with non-Chinese. The above US and Singaporean studies suggest that ethnicity is a key characteristics of HALE in multicultural societies in developed countries (16). In developing countries, the health expectancy differential across ethnicities has not been verified yet.

The Guangxi Zhuang Autonomous Region in southern China is an ethnically diverse region containing 11 ethnic minority groups, including the Zhuang, Yao, Miao, Dong, Mulao, Maonan, and Jing minorities, the Han majority group, and so on. The region is one of the four regions with a high centenarian ratio in China, where LE at birth increased from 71.2 years in 2000 to 75.1 years in 2010 (21). The diversity of ethnic cultures is completely preserved at present because of minority residential segregation. Although previous data suggested that certain subgroups, such as the Jing and Yao minority groups, attain good longevity, few data are available on the extent of such disparities in HE among subgroups and whether those living longer suffer more related-age problems and loss in years of life. Thus, we conducted this study with the objective of assessing the extent of disparities in HE among the elderly from different ethnic groups using the QALE method.

## Subjects and methods

### Study subjects and sampling techniques

The methods of sampling and selection of the subjects were described in a previously published article (22). In brief, this was a community-based cross-sectional survey among eight ethnic groups in the Guangxi Zhuang Autonomous Region of China. A total of 126 villages were selected and the 6,998 eligible elderly aged 60 years and older were randomly selected from the relevant village administrative committee.

### HRQoL measurement

The HRQoL of the elderly was evaluated using a Chinese version of the EQ-5D-3L instrument, which is a standardized HRQoL questionnaire developed by the EuroQoL Group in 1990 (23). The Chinese version of the EQ-5D-3L instrument for general populations has demonstrated acceptable construct validity and test–retest reliability (24). The methods for collecting data were described in more detail elsewhere (22).

The respondents objectively evaluated their current health status based on the five dimensions of the EQ-5D questionnaire (mobility, self-care, usual activities, pain/discomfort, and anxiety/depression), with three response levels (no problems, some or moderate problems, extreme problems). The health profile from the EQ-5D theoretically results in 243 unique health states. Finally, the weighted EQ-5D index values (also called *utility values*) were derived by applying the scoring algorithm of the general Chinese population sample. The algorithm was developed by Liu using the time trade-off method (9). The index values as an aggregated utility index (UI) were calculated using the following formula:

$$U1=1-0.039-(\text{M}O_{2}\times 0.099)-(\text{M}O_{3}\times 0.246)-(SC_{2}\times 0.105)-(SC_{3}\times 0.208)-(UA_{2}\times 0.074)-(UA_{3}\times 0.193)-(PD_{2}\times 0.092)-(PD_{3}\times 0.236)-(AD_{2}\times 0.086)-(AD_{3}\times 0.205)-(N_{3}\times 0.022)$$

where MO, SC, UA, PD, and AD represent the mobility, self-care, usual activities, pain/discomfort, and anxiety/depression dimensions, respectively. The subscripts 2 and 3 represent the response levels: Level 2 indicates some or moderate problems, and Level 3 indicates extreme problems; N_3_ is equal to 1 if any dimension is at Level 3; otherwise it is 0. The index values require valuations for all relevant health states on a scale anchored at 1 (full health) and 0 (dead).

### Population and mortality data at county level

Age-specific population and mortality data at county level in Guangxi Province were obtained from the Sixth National Population Census of China, published in the Statistical Yearbook in 2012 (25). The under-enumeration rate of the census was lower than those in recent censuses by some developed countries (26).

### LE and QALE calculation

LE at birth was calculated on the basis of the population and mortality data for 5-year age bands at county level in this study. For each county, these were used to construct abridged life tables (0, 1, 1–4, 5–9, …, 85+ years) using an adaptation of Chiang's method to obtain estimates of LE (27). Because participants in this survey were aged 60 years and older, only the life expectancies for this age group were relevant. Additionally, QALE at the age of 60 years (QALE_60_) was estimated for each ethnicity because states of health vary considerably among ethnic groups. The subgroup sample of each ethnic group was selected from the specific ethnic autonomous administrative county. Consequently, LE at the age of 60 years (LE_60_) at county level can represent the value of LE for each ethnic group. The age-specific UI values (UI in Equation 1) for each ethnic group were calculated based on the results of the EQ-5D questionnaire.

The quality-adjusted survival for the elderly of each ethnic group was established by integrating the HRQoL with the survival function (survival probability from the life table) at each age-specific point (28). The difference between quality-adjusted survival and survival probability can be used to assess health risk, quantify changes in or loss of LE, and burden of disease for different health conditions.

However, we could not directly estimate QALE_60_ on the basis of quality-adjusted survival because age-specific population and mortality data in the National Population Census were limited. The HRQoL utility values were combined with the LE data by using Sullivan's method to estimate QALE_60_ for each ethnicity (29). The formula for calculating QALE_60_ is given by the following:

$${e^{\prime }}_{x}=\frac{1}{l_{x}}\sum_{x=0}^{w}{\left(1{-}_{n}\pi_{x}\right)}_{n}L_{x}$$

where *l*_*x*_ is the number of survivors at the exact age *x*; _*n*_φ_*x*_ represents the prevalence of a determined state of health among individuals with age in the interval (*x*, *x*+*n*); _*n*_*L*_*x*_ is the total number of years lived by a cohort in age group (*x*, *x*+*n*); and *w* represents the maximum age.

### Ethical considerations

The protocol of this study was approved by the Ethics Committee of the Faculty of Medicine, Prince of Songkla University, Hat Yai, Songkhla Province, Thailand (reference number 57-188-18-5) before the research was carried out.

## Results

### Characteristics of respondents

In total, 6,511 eligible participants agreed to join the survey. The response rate was 93%. The demographic characteristics of the respondents by ethnic group are presented in Table 1. The maximum age of the respondents was 105 years. The age distribution of each ethnic group was similar; however, ethnic Jing people were slightly older. Females outnumbered males in all ethnic groups.

**Table 1:** Age and gender distribution of the elderly among all ethnic groups

	Ethnic group								
		
Variables	Zhuang	Dong	Mulao	Han	Maonan	Miao	Yao	Jing	Overall

*N*	1,588	919	840	826	823	664	449	402	6,511
Age group (%)									
60–64	25.4	25.4	25.2	31.2	27.1	30.3	28.5	17.2	26.5
65–69	17.5	23.5	14.6	17.3	17.3	19.1	21.6	11.7	18.0
70–74	19.4	21.1	20.7	19.5	21.4	20.3	22.3	17.7	20.3
75–79	15.6	13.2	16.3	14.8	13.0	13.4	13.4	22.1	14.9
80–84	13.4	10.9	14.2	9.6	13.2	11.3	9.4	18.2	12.4
≥85	8.7	6.0	8.9	7.6	8.0	5.6	4.9	13.2	7.8
Age mean (SD), years	71.9 (8.7)	70.6 (7.9)	72.1 (8.7)	70.7 (8.6)	71.2 (8.6)	70.3 (8.2)	70.3 (7.8)	74.7 (8.9)	71.3 (8.5)
Gender (%)									
Male	47.2	49.7	45.6	48.9	44.5	48.9	48.3	44.5	47.3
Female	52.8	50.3	54.4	51.1	55.5	51.1	51.7	55.5	52.7

### Age and gender difference in HRQoL across ethnic group

**Fig. 1:**
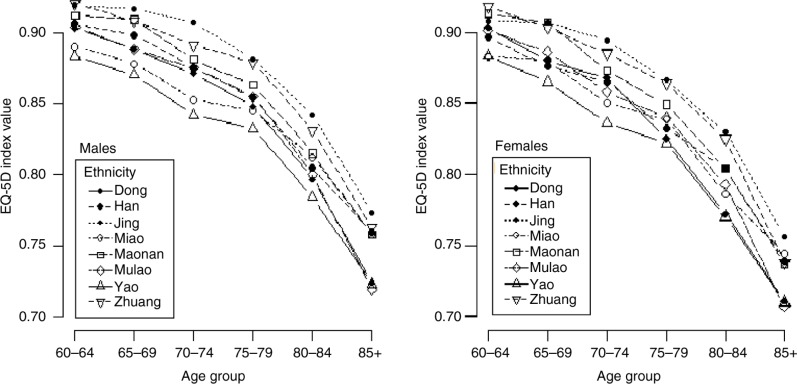
European Quality of Life five-dimension index utility values by ethnicity, age, and gender.

Figure 1 shows the average HRQoL utility value by age and gender across ethnic groups. The HRQoL utility value of the elderly for all ethnic groups was 0.848 (95% CI: 0.868–0.827) in men and 0.832 (95% CI: 0.849–0.814) in women. The value generally decreased with increasing age, was highest in those of Jing ethnicity, and lowest in those of Yao ethnicity. Men had a higher overall HRQoL utility value than women.

### Probability of surviving and quality-adjusted survival

**Fig. 2:**
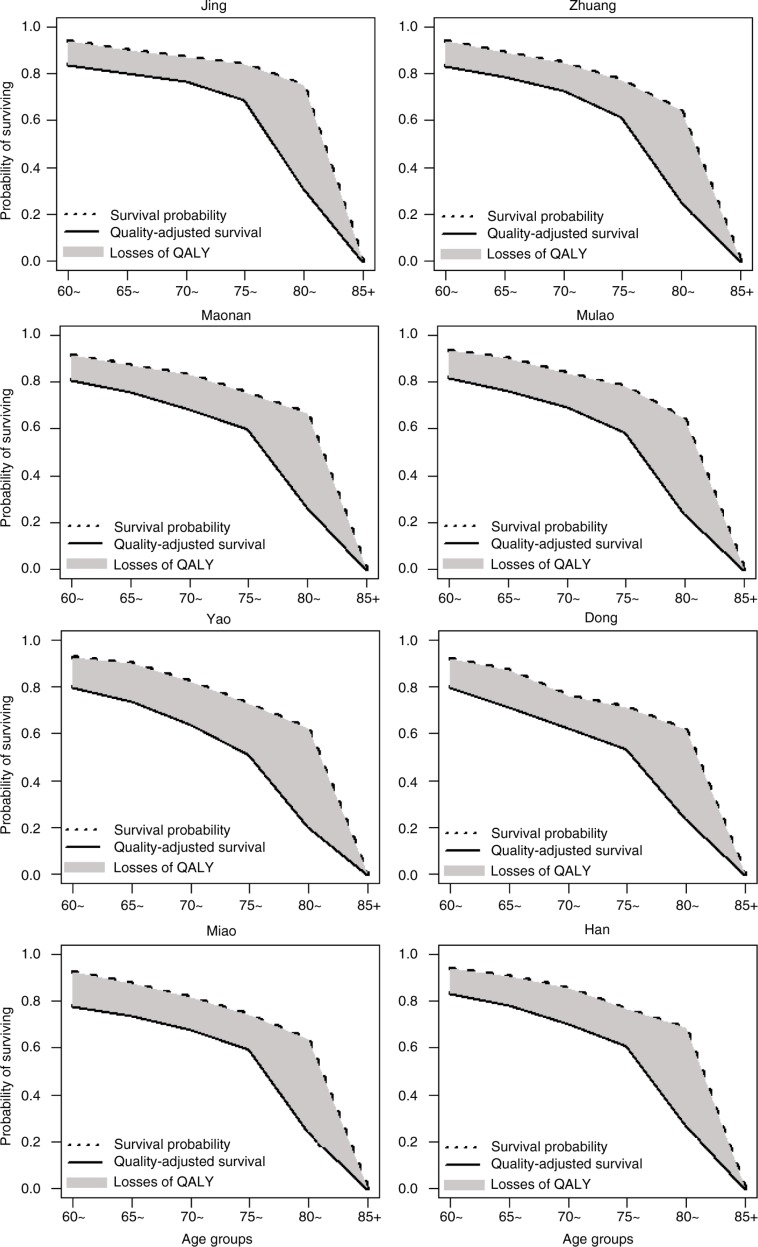
Probability of surviving and quality-adjusted survival for males.

**Fig. 3:**
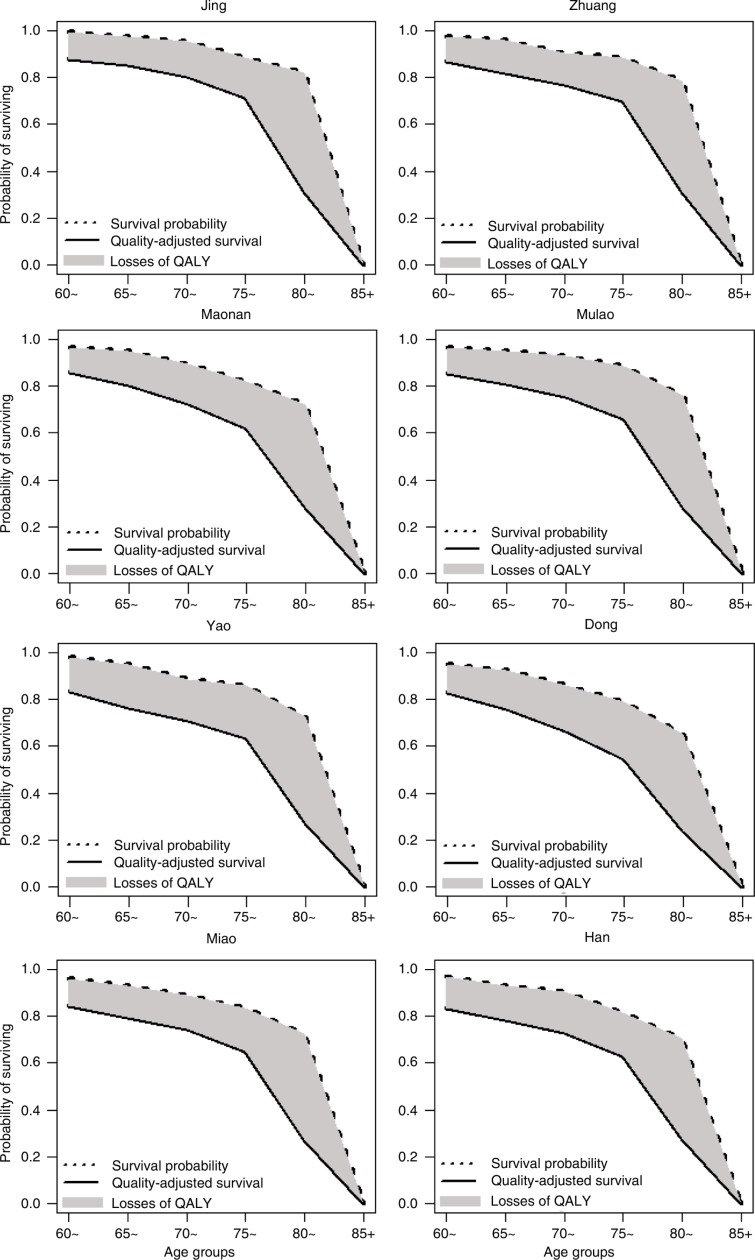
Probability of surviving and quality-adjusted survival for females.

Survival probability and quality-adjusted survival by gender for each ethnic group are illustrated in Figs. 2 and 3, respectively. Survival probability and quality-adjusted survival of the elderly gradually decreased with increasing age, especially the elderly aged 75 years and over. Participants from the Jing, Zhuang, and Mulao ethnic groups scored higher in both survival probability and quality-adjusted survival than others. Loss of quality-adjusted life years (QALYs) (shaded area between the two probability curves) was higher for the Yao and Dong groups than for the others. Women had higher survival probability than men, but their QALY loss was higher.

### LE, QALE, and QALY loss among ethnic groups

**Fig. 4:**
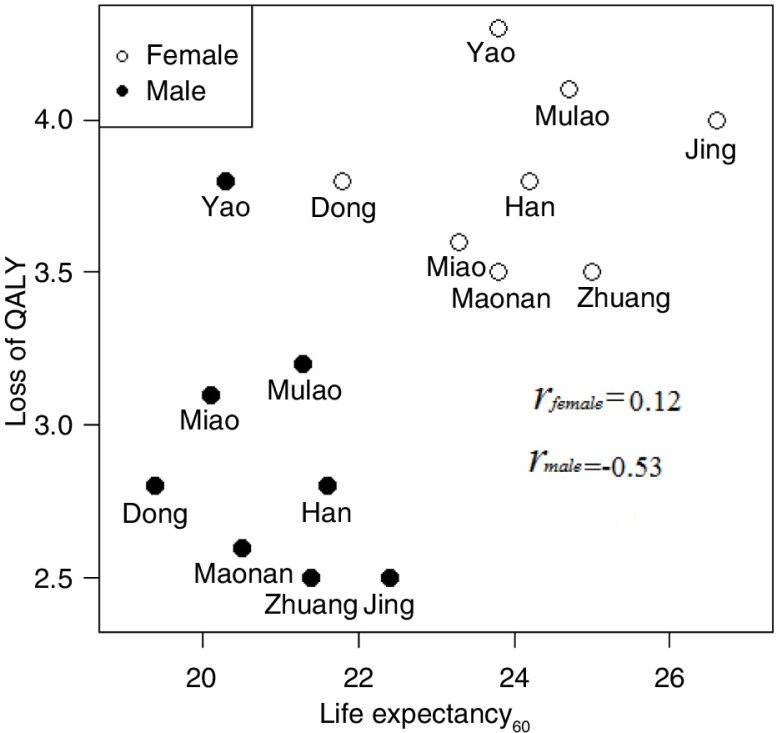
Scatter plot between life expectancy at age 60 and losses of quality-adjusted life years for both males and females.

The overall LE at birth was 77.7 (SD=5.5) years among all participants; the minimum LE at birth was 75.1 (among the Dong) and the maximum was 80.9 (among the Jing). LE_60_, QALE_60_, and QALY loss for all ethnic groups were 20.9 (95% CI: 21.7–20.0), 18.0 (95% CI: 18.9–16.9), and 2.9 (95% CI: 3.3–2.5) years in males, respectively, and 24.2 (95% CI: 25.3–23.0), 20.3 (95% CI: 21.5–19.2), and 3.8 (95% CI: 4.1–3.6) years in females (Table 2). The average QALY loss for men was 2.9 years and 3.8 years for women. The maximum QALY loss among all ethnic groups was 3.8 years in males and 4.3 years in females. Figure 4 shows QALYs lost plotted against LE_60_ for both males and females across the eight ethnic groups. Females had a higher LE_60_ and years of QALYs lost compared to males. Within the same sex, the correlation between LE and QALY loss was −0.53 in males and 0.12 in females. In other words, among men, ethnic groups with higher longevity tended to have less QALY loss. However, this trend was not observed in women.

**Table 2:** LE_60_, QALE_60_, and QALY loss by gender for the eight ethnic groups

Ethnic group	LE_60_	QALE_60_	QALY loss	QALY loss (%)
Men (mean±SD)	20.9±1.0	18.0±1.2	2.9±0.4	14.0±2.4
Jing	22.4	19.9	2.5	11.0
Han	21.6	18.8	2.8	12.8
Zhuang	21.4	18.9	2.5	11.7
Mulao	21.3	18.1	3.2	15.1
Maonan	20.5	17.9	2.6	12.8
Yao	20.3	16.5	3.8	18.6
Miao	20.1	17.0	3.1	15.3
Dong	19.4	16.6	2.8	14.4
Women (mean±SD)	24.2±1.4	20.3±1.4	3.8±0.3	15.9±1.4
Jing	26.6	22.6	4.0	14.9
Han	24.2	20.4	3.8	15.7
Zhuang	25.0	21.6	3.5	13.9
Mulao	24.7	20.6	4.1	16.6
Maonan	23.8	20.2	3.5	14.9
Yao	23.8	19.5	4.3	18.0
Miao	23.3	19.7	3.6	15.4
Dong	21.8	18.0	3.8	17.6

LE_60_, life expectancy at age 60; QALE_60_, quality-adjusted life expectancy at age 60; QALY, quality-adjusted life year.

## Discussion

HRQoL among elderly people from different ethnic groups generally declined with increasing age, and women were more likely to report worse health status than men. Although women had a higher probability of surviving than men in later life, QALY loss in women was also higher. More importantly, the disparity in survival among ethnic groups was more remarkable when quality of life was taken into account. Among men, ethnic groups with higher longevity tended to have higher QALYs; however, this was not observed in women.

Women were more likely to report worse health status than men, which is consistent with the results of previous studies (13, 30, 31). The gender differences in HRQoL among the elderly might be explained by differences in performance-based functional capacity and chronic conditions. The worse HRQoL in women is probably due to a higher prevalence of disability and chronic conditions, particularly arthritis and depression (32). In addition, women suffer from more age-related problems in later life such as depression, loneliness, and empty-nest syndrome, compared with men. The EQ-5D instrument used to assess the HRQoL of the elderly in this study is more likely to capture the problems and symptoms in females (33). These results suggest that it is essential to take more care of rural elderly women and to reduce the gender inequity gap in health status.

Disparities in terms of HRQoL between ethnic groups were observed in the present study, which is consistent with previous studies from the United Kingdom (34, 35), United States (36), and Singapore (37). When interpreting the results of disparities between ethnic groups, the minimum important differences (MID) in health status need to be addressed. The maximum difference in the HRQoL utility value between ethnic groups was 0.045, which is 1.5 times as high as the MID of 0.03 in health status (38). The causes for such diversity could be explained by race-based residential segregation as a fundamental cause of racial disparities in health. The majority of the elderly reside in mountainous regions or regions bordering Vietnam (e.g., the Yao, Miao, and Dong ethnic groups), which has led to relatively high physical separation and the development of special ethnic regional culture. Previous studies concluded that residential segregation is a primary cause of racial differences in socioeconomic status, which in turn remains a fundamental cause of racial differences in health (39, 40). The spatial distribution of ethnic groups contributes to disparities in health by influencing access to social, economic, and physical resources essential to health. Future studies are needed to examine the relationship between residential segregation and the health status of a population across ethnic groups in order to obtain good explanations for such variance in HRQoL.

In the present study, disparity in survival among ethnic groups was more remarkable when quality of life was taken into account. The disparity in survival may be accentuated by differences in overall health across ethnicities. Quality-adjusted survival is based not only on survival probability but is also adjusted for time spent with poor health status. In other words, quality of life was able to capture the overall health status of a population associated with both mortality and morbidity. In this study, ethnic groups with high quality-adjusted survival (such as the Jing, Zhuang, and Han) not only had high survival probability but also reported better physical functioning and subjective well-being. However, most of the Yao, Mulao, Miao, and Dong people live in rural mountainous areas with poor infrastructure and health care services, which might be more likely to result in higher disability and morbidity. Such disparity in quality of life is a concern, and steps should be taken to reduce the gap between ethnic groups in the future.

Our finding in men, but not in women, that ethnic groups with a higher longevity tended to have higher QALY is inconsistent with previous studies from Western countries (41) and other parts of China (42). The disparity might partly be attributed to differences in physical activity levels, lifestyle choices, and folk culture among different ethnic groups. Traditionally, alcohol consumption and cigarette smoking are more likely to be accepted among Chinese men, especially in some ethnic groups such as the Mulao and Dong (43). Studies found that unhealthy lifestyle behaviors in males such as total energy, fat, protein, and salt intakes were higher among the Han than in other ethnic groups, whereas physical activity level, vegetal protein, and total dietary fiber intakes were lower among the Han (44). However, fish sauce and glutinous rice mixed with sesame are the favorite condiment and main food of the Jing minority, contributing to a healthy lifestyle and having longevity benefits. In addition, minority fork culture, as a macro component, is an important contextual determinant of HRQoL (45). Ethnic culture for the elderly might enhance their beliefs and activities to improve HRQoL (46–48). For example, antiphonal songs in the Jing ethnic group, which are listed in the intangible cultural heritage of China, are an important part of traditional ethnic folk culture. Most elderly Jing are followers of Buddhism or Taoism and thus keep to a special vegetarian diet. In general, elderly men are more likely to perform outdoor physical activities while participating in festivals, folk sports, and intangible cultural heritage, which may help them to maintain their capacity for daily living, whereas women are more likely to stay indoors (42). However, the relationship between traditional ethnic folk culture and the health outcomes of a population is unclear. Future studies are needed to examine the differences in ethnic cultures, lifestyle choices, religious beliefs, dietary habits, and social circumstances that influence quality of life in order to obtain a good explanation for such variation.

## Limitations

Mortality and population data from the Sixth National Population Census of China was collected in 2010, but data on the HRQoL in this survey were collected in 2014. However, the 4-year time lag is unlikely to influence LE_60_. Thus, it is unlikely to introduce remarkable bias on the differences of QALY.

## Conclusions

Women live longer than men, but they suffer more; men have a shorter life expectancy, but those who live longer are healthier. Attempts should be made to reduce suffering in elderly women and to improve the longevity of men. Certain ethnic groups, such as the Dong, Yao, and Miao, had low levels of QALE. They would benefit from more health promotion measures including lifestyle improvement and access to health care services.

## Paper context

Ethnic culture and gender are important determinants of the health status of a population. In multicultural societies, however, the dominant approaches for assessing the health status of the elderly have ignored differences based on gender and ethnicity. This paper estimates quality-adjusted life expectancy for the elderly from different ethnic groups and highlights that attempts should be made to reduce suffering among women, improve the longevity of men, and provide more health promotion measures for certain ethnic minorities.
